# The X-Rays as an Aid in the Early Diagnosis of Pulmonary Tuberculosis*Read before the International Society of Mexico, Section in Electro-Therapeutics, January 24, 1908.

**Published:** 1908-03

**Authors:** R. H. L. Bibb

**Affiliations:** Saltillo


### TEXAS MEDICAL JOURNAL.

Established July, 1885.

F. E. DANIEL, M. D., - - - - Editor, Publisher and Proprietor

PUBLISHED MONTHLY.—SUBSCRIPTION $1.00 A YEAR.

VOL. XXIII. AUSTIN, MARCH, 1908. No. 9.

The publisher is not responsible for the views of contributors.

For the Texas Medical Journal.

### The X-Rays as an Aid in the Early Diagnosis of Pulmonary Tuberculosis.*

BY R. H. L. BIBB, M. D., SALTILLO.

Roentgen discovered the X-Rays in the latter part of the year 1895, and in October, 1896, Williams, of Boston, published, in the *Boston Medical and Surgical Journal,* that "I have examined about forty cases of pulmonary tuberculosis, and find not only that the fluoroscope is of value in determining the extent of the disease, but also sometimes reveals its location where and when it would otherwise have been unsuspected." Bouchard, in December of the same year, gave an account of the X-Rays in the diagnosis of pulmonary tuberculosis. So it will be seen that to Williams is due the credit of calling the attention of the medical profession to this invaluable aid in diagnosing a disease, upon the early recognition of which depends the welfare of every one of its millions of victims. Following the announcement made by Williams, the subject was taken up in all parts of the civilized world, and the value of the procedure is recognized, today, as one of the most valuable that the physician can call to his aid in the early diagnosis of pulmonary tuberculosis.

It will be noted that the word "aid" has been used in the preceding paragraph. This has been done advisedly, for no one will contend that a diagnosis can be made, in all cases, by the X-Rays, apart from the history; but it is asserted, and it is recognized, everywhere, that there are one, or more, symptoms, common to all

*Read before the International Society of Mexico, Section in Electro-Therapeutics, January 24, 1908.

cases of pulmonary tuberculosis, in its primary stage, that can be recognized by the X-Rays, and which can not be recognized in any other way, at the present time. Four of these symptoms, in the order of their value, are the following:

1. The movements of the diaphragm—first pointed out by Williams—is restricted on the affected side or sides, usually in the lower part of its excursion.

2. One or both apices of the lungs fail to light up on deep inspiration.

3. The diseased portion of the lungs casts a shadow on the screen or fluoroscope.

4. The heart, in a large majority of cases, is smaller than normal, and is placed more vertically in the chest.

Of these changes or symptoms, the restricted movements of the diaphragm is one of the earliest and most frequent signs, and in many cases may be discovered long before failure of the lung to light up becomes manifest. This restricted movement of the diaphragm is admitted by all observers; but they are not agreed that the sign precedes other signs, in all cases. Williams says it is one of the very earliest signs of pulmonary tuberculosis. Bonnet Leon writes that the "diaphragmatic sign" occurs in the "pretuberculous period" of the disease, and he claims that children and adults of tuberculous parents nearly all show it. There is no consensus of opinion as to the cause of this valuable and interesting symptom. It has been attributed to:

1. Toxic cause, impairing tissue vitality.
2. Loss of elasticity in the lung tissue.
3. Unrecognizable pleuritic adhesions.
4. Faulty reflex innervation, etc.

As you all know, when a healthy lung is lighted up by an X-Ray tube, it is clear and spotless from apex to base. When examined with the fluoroscope there is a pronounced difference in its fluorescence at the end of deep inspiration and expiration. On deep inspiration the area of fluorescence as shown by the fluoroscope is largely increased, due, according to Williams, to a lesser quantity of blood in the lung, but, according to the opinion of others, due to a distension of the lungs by air which makes them more permeable to the rays. Walsham and Orton say that, "The failure to brighten up of one or other or both apices on deep inspiration is one of the earliest signs of pulmonary tuberculosis and can certainly be made out before any distinct mottled shadowing can be detected." They recommend that "in making a comparison between the two apices, care must be taken to adjust the light so that the suspected side is barely illuminated, as the contrast between it and the normal side will then be more obvious. If the quantity of rays employed or their penetrating powers be too great, small difference in density will not be apparent, and the condition entirely overlooked." In difficult cases they recommend placing a lead diaphragm between the patient and tube.

The shadows cast upon the fluoroscope in the early stage of pulmonary tuberculosis is due to lung consolidation, and when the foci of such consolidation are small and scattered, they may be discovered by the X-Ray before their discovery is at all possible by any other method of examination.

Walsham and Orton assert that "we have frequently noticed that in a large majority of cases of pulmonary tuberculosis the heart is seen to be much smaller than normal, and placed more vertically in the chest. In some cases the extreme smallness of this organ is very striking." Bouchard and Baltharzard, of Paris, claim that the heart, in the first stage of pulmonary tuberculosis, is smaller than normal, and regard the reduced size of the organ as characteristic of "a tuberculous soil"—one of the dystrophies which predisposes to tuberculosis. Guilleminot believes that cases of tuberculosis which are cured are those who possessed hearts larger than the normal size. The size of the heart and its position can now be determined with a percision not attainable before the introduction of the X-Rays and the orthodiagraph in chest examinations; and it is in this connection that the X-Rays loan such valuable aid in making a diagnosis in an obscure case of pulmonary tuberculosis, when a knowledge of the size and position of the heart is necessary to that end.

To return to the subject of restricted movements of the diaphragm, it may be well to give conclusions of Guilleminot on its importance, since he has studied it from every standpoint. His conclusions are as follows:

"1. That on the right side the mean position of the diaphragmatic curve is 16.5 centimeters below the supra sternal notch, and on the left side 18.5 centimeters below that line.

2. The normal amplitudes of the diaphragmatic incursion is from 16 to 18 millimeters; it is approximately equal on the two sides.

3. Any variation in the amount of incursion on the right and left sides is a pathological symptom."

In concluding this fragmentary writing, the following hypothetical case is submitted—a case not altogether hypothetical in the experience of very many practitioners:

A young lady, aged 18, returns from school complaining of general weakness, anemia, loss of flesh, loss of appetite, an evening temperature of 37.2, a morning temperature of 36.8. No cough, no expectoration, no hemoptysis, no night sweats. Physical examination absolutely negative. An X-Ray examination shows an almost imperceptible shadow of the left apex down to third rib.

DIAPHRAGMATIC MOVEMENTS.

Quiet respiration, right side, five-eighths inch; left side, one-half inch; forced respiration, right side, two and three-quarter inches; left side, two and one-half inches.

With this data in hand, it is submitted that a positive diagnosis of incipient pulmonary tuberculosis of the left lung can be made, and that it can not be made by any other process in use at the present day.

